# Disease Surveillance Among U.S.-Bound Immigrants and Refugees — Electronic Disease Notification System, United States, 2014–2019

**DOI:** 10.15585/mmwr.ss7102a1

**Published:** 2022-01-21

**Authors:** Christina R. Phares, Yecai Liu, Zanju Wang, Drew L. Posey, Deborah Lee, Emily S. Jentes, Michelle Weinberg, Tarissa Mitchell, William Stauffer, Julie L. Self, Nina Marano

**Affiliations:** ^1^Division of Global Migration and Quarantine, National Center for Emerging and Zoonotic Infectious Diseases, CDC; ^2^Departments of Medicine and Pediatrics, Center for Global Health and Social Responsibility, University of Minnesota; ^3^Division of Tuberculosis Elimination, National Center for HIV, Viral Hepatitis, STD, and TB Prevention, CDC

## Abstract

**Problem/Condition:**

Each year, approximately 500,000 immigrants and tens of thousands of refugees (range: 12,000–85,000 during 2001–2020) move to the United States. While still abroad, immigrants, refugees, and others who apply for admission to live permanently in the United States must undergo a medical examination. This examination identifies persons with class A or B conditions. Applicants with class A conditions are inadmissible. Infectious conditions that cause an applicant to be inadmissible include infectious tuberculosis (TB) disease (class A TB), infectious syphilis, gonorrhea, and infectious Hansen’s disease. Applicants with class B conditions are admissible but might require treatment or follow-up. Class B TB includes persons who completed successful treatment overseas for TB disease (class B0), those with signs or symptoms suggestive of TB but whose overseas laboratory tests and clinical examinations ruled out current infectious TB disease (class B1), those with a diagnosis of latent TB infection (LTBI) (class B2), and the close contacts of persons known to have TB disease (class B3). Voluntary public health interventions might also be offered during the overseas examination. After arriving in the United States, a follow-up TB examination is recommended for persons with class B TB.

**Period Covered:**

This report summarizes health information that was reported to CDC’s Electronic Disease Notification (EDN) system for refugees, immigrants, and eligible others who arrived in the United States during 2014–2019. Eligible others are persons who although not classified as refugees (e.g., certain parolees, special immigrant visa holders, and follow-to-join asylees) are eligible for the same services and benefits as refugees.

**Description of System:**

The EDN system has both surveillance and programmatic components. The surveillance component is a centralized database that collects 1) health-related data from the overseas medical examination for immigrants with class A or B conditions and for all refugees and eligible others and 2) TB-related data from the postarrival TB examination. The programmatic component is a reporting system that sends arrival notifications to state and local health agencies in the jurisdiction where newly arriving persons have reported intending to live and provides state and local health agencies and other authorized users with medical data from overseas examinations.

**Results:**

During 2014–2019, approximately 3.5 million persons moved to the United States from abroad, including 3.2 million immigrants, 313,890 refugees, and 95,993 eligible others. Among these, the overseas examination identified 139,683 persons (3,903 per 100,000 persons examined) with class B TB, 54 with primary or secondary syphilis (30 per 100,000 persons tested), 761 with latent syphilis (415 per 100,000 persons tested), and, after laboratory testing for gonorrhea was added in 2016, a total of 131 with gonorrhea (374 per 100,000 persons tested). Refugees were offered additional, voluntary interventions, including vaccinations and presumptive treatment for parasites. By 2019, first- and second-dose coverage with measles-containing vaccine were 96% and 80%, respectively. In refugee populations for whom presumptive treatment is recommended, up to 96% of refugees, depending on the specific regimen, were offered and accepted treatment. For the 139,683 persons identified overseas with class B TB, EDN sent arrival notifications and overseas medical data to the appropriate state or local health agency to facilitate postarrival TB examinations. Among 101,119 persons identified overseas as having class B0 TB (6,586) or class B1 TB (94,533), a total of 67,432 (67%) had a complete postarrival examination reported to EDN. Among 35,814 children aged 2–14 years identified overseas with class B2 TB, 20,758 (58%) had a complete postarrival examination reported to EDN. (Adults are not routinely tested for immune reactivity to *Mycobacterium tuberculosis* during the overseas medical examination.) Among those with a complete postarrival examination reported to EDN, the number with a diagnosis of culture-positive TB disease within the first year of arrival was 464 (688 cases per 100,000 persons examined) for those with class B0 or B1 TB and was 11 (53 cases per 100,000 persons examined) for children with class B2 TB.

**Interpretation:**

During 2014–2019, the overseas medical examination system prevented importation of 6,586 cases of infectious TB, 815 cases of syphilis, and 131 cases of gonorrhea. When the examination is used to offer public health interventions, most refugees (up to 96%) accept the intervention. Postarrival follow-up examinations, which were completed for 88,190 persons and identified 475 cases of culture-positive TB, represent an important opportunity to further limit spread of TB disease in the United States by identifying and providing, if needed, preventive care for those with LTBI or treatment for those with disease.

**Public Health Action:**

Federal, state, and local health departments and agencies should continue to use EDN data to monitor, evaluate, and improve health-related programs and policies aimed at U.S.-bound or recently arrived immigrants, refugees, and eligible others. Additional public health interventions that could be offered during the overseas medical examination should be considered (e.g., treatment for LTBI). Finally, for persons with class B TB, measures should be taken to identify and remove barriers to completing postarrival examinations to reduce risk for TB disease and community transmission, along with measures to encourage reporting of completed examinations for better data-driven decision-making.

## Introduction

### Background

Each year, approximately 500,000 immigrants and tens of thousands of refugees and eligible others move to the United States after applying for admission while overseas (any country other than the United States and its territories). Eligible others are persons who although not classified as refugees (e.g., certain parolees, special immigrant visa holders, and follow-to-join asylees) are eligible for the same services and benefits as refugees. As part of the admission process, these immigrants, refugees, and eligible others undergo an overseas medical examination to determine medical admissibility. The U.S. Department of Health and Human Services (HHS) has the regulatory authority to require this examination (*1*–*4*) and describes these requirements in technical instructions issued by CDC. As of 2019, the examinations were conducted by approximately 600 licensed panel physicians appointed by the U.S. Department of State (DOS), working in 350 clinics in 160 countries.

The overseas medical examination identifies applicants with class A and class B conditions (Box 1). An applicant with a class A communicable disease cannot be admitted to the United States until the disease has been successfully treated or the U.S. Department of Homeland Security (DHS) grants a waiver. When a class A condition resolves, the applicant is reclassified as having a class B condition and is allowed to travel to the United States. The U.S. Code of Federal Regulations specifies four class A communicable diseases of public health significance: tuberculosis (TB) disease, infectious syphilis, gonorrhea, and infectious Hansen’s disease. Persons with class B conditions can be admitted; however, the condition might require treatment or follow-up. Persons with class B TB include those who have successfully completed treatment overseas for TB disease (class B0), those with signs or symptoms suggestive of TB disease but whose overseas laboratory tests and clinical examinations ruled out current infectious TB disease (class B1), those with a diagnosis of latent TB infection (LTBI) (class B2), and the close contacts of persons known to have TB disease (class B3).

BOX 1 **Tuberculosis, syphilis, gonorrhea, and Hansen’s disease classifications assigned during the overseas medical examination for persons seeking admission to the United States**

No tuberculosis (TB) classification*

This class includes applicants without current clinical findings of TB disease, without known HIV infection, and with a normal chest radiograph (and for applicants who require it, a negative interferon gamma-release assay [IGRA]) with normal TB disease screening examinations.

**Class A TB disease**

This class includes all applicants who have TB disease. This class also includes applicants with extrapulmonary TB who have a chest radiograph suggestive of pulmonary TB disease, regardless of sputum smear and culture results.

**Class B0 TB, pulmonary^†^**

This class includes applicants with TB diagnosed by the panel physician or who were seen by the panel physician while receiving TB treatment and successfully completed CDC-defined directly observed treatment (DOT) under the supervision of a panel physician before immigration.

**Class B1 TB, pulmonary^†^**

This class includes applicants who have signs or symptoms, physical examination, or chest radiograph findings suggestive of TB disease or who have known HIV infection but negative acid-fast bacillus sputum smears and negative cultures and do not have diagnosed TB disease. This classification also includes applicants who have TB disease diagnosed by the panel physician, have refused DOT treatment, and are returning after treatment and completion of 1-year wait.

**Class B1 TB, extrapulmonary**

This class includes applicants with diagnosed extrapulmonary TB who have a normal chest radiograph and negative sputum smears and negative cultures.

**Class B2 TB, latent TB infection (LTBI) evaluation**

This class includes applicants who have a positive IGRA or tuberculin skin test (TST) but otherwise have a negative evaluation for TB. Contacts with a positive IGRA or TST of ≥5 mm induration must receive this classification in addition to a classification of class B3, contact evaluation (if they are not already classified as class B0 TB, pulmonary; class B1 TB, pulmonary; class B1 TB, extrapulmonary; or class A TB).

**Class B3 TB, contact evaluation**

This class includes applicants who are a recent contact of a person with known TB disease, regardless of IGRA or TST results. If the IGRA or TST results are positive and no evidence of TB disease exists, the applicant has two classifications: class B2 and class B3; if results are negative, only class B3 applies.

**Class A for syphilis, gonorrhea, or Hansen’s disease**

This class includes applicants with untreated syphilis, gonorrhea, or Hansen’s disease.

**Class B for syphilis, gonorrhea, or Hansen’s disease**

This class includes applicants who completed treatment for syphilis and gonorrhea or at least 1 week of therapy for Hansen’s disease.

**Source:** CDC. Tuberculosis technical instructions for panel physicians. Atlanta, GA: US Department of Health and Human Services, CDC. https://www.cdc.gov/immigrantrefugeehealth/exams/ti/panel/tuberculosis-panel-technical-instructions.html

* No TB classification means that TB disease was ruled out at the time of the examination but does not necessarily mean that LTBI has been ruled out because testing for immune reactivity to *Mycobacterium tuberculosis* is not required for most adults. On the basis of LTBI prevalence in their countries of origin, many adults who meet criteria for no TB classification likely have LTBI.

^†^ Before October 1, 2018, applicants who met criteria for class B0 or B1 TB, pulmonary, were aggregated, with both referred to as class B1 TB, pulmonary. This report uses the disaggregated classifications, determined retroactively, throughout.

In addition to the four specified conditions, other class A communicable diseases include quarantinable communicable diseases; these diseases are designated by a presidential executive order and are, currently, infectious TB, cholera, diphtheria, measles, plague, smallpox, yellow fever, viral hemorrhagic fevers, severe acute respiratory syndromes (COVID-19, Middle East respiratory syndrome, and severe acute respiratory syndrome [SARS], and influenza caused by novel or reemergent influenza) (*5*), and communicable diseases posing a public health emergency of international concern (PHEIC) (*6*) that could be imported into the United States and affect U.S. residents. With the exception of infectious TB, data about quarantinable and PHEIC conditions are not systematically captured by the routine overseas medical examination data collection process; when such conditions are present, routine processes give way to emergency response measures, which might include requirements for predeparture vaccinations, additional testing, isolation and quarantine, or suspension of processing and travel. This report focuses on TB, syphilis, gonorrhea, and Hansen’s disease.

The overseas examinations are used as opportunities to offer additional, voluntary public health interventions. Major interventions offered to refugees include a vaccination program for most vaccine-preventable diseases and a presumptive treatment program for soil-transmitted helminthiasis, strongyloidiasis, schistosomiasis, and malaria infection. 

A postarrival TB examination is recommended by CDC for immigrants who have class A TB (and are admitted with a waiver) or class B TB. A comprehensive postarrival examination that includes a TB examination is recommended by CDC for all refugees and eligible others.

#### Overseas Medical Examination

In accordance with CDC Technical Instructions for Panel Physicians (*7*), the overseas medical examination for all applicants must include a medical history and physical examination. Additional required procedures and tests to further screen for TB, syphilis, gonorrhea, and Hansen’s disease depend on age and location (Box 2). Panel physicians document their findings on the following DOS forms: DS-2054 Medical Examination for Immigrant or Refugee Applicant, DS-3030 TB Worksheet, DS-3025 Vaccination Documentation Worksheet, and DS-3026 Medical History and Physical Examination Worksheet. Medical examinations are valid for no more than 6 months. CDC ensures that these examinations fulfill the requirements through a robust quality assurance program that includes site visits, evaluations, and trainings.

BOX 2 **Overseas screening requirements for tuberculosis, syphilis, gonorrhea, and Hansen’s disease for persons seeking admission to the United States**

**Tuberculosis (TB)**

In countries with a World Health Organization (WHO)-estimated TB incidence of ≥20 cases per 100,000 population, applicants aged 2–14 years must receive an interferon-gamma release assay (IGRA) and, if the result is positive, chest radiography. In countries with <20 cases per 100,000 population, applicants aged <15 years who have signs or symptoms suggestive of TB disease and those who disclose HIV infection must receive an IGRA and chest radiography. (Testing for HIV infection is not required.) Before October 1, 2018, tuberculin skin tests were also allowed as an alternative to IGRAs. In all countries, regardless of TB incidence, applicants aged ≥15 years must have chest radiography, and applicants of any age who have signs, symptoms, or chest radiographs suggestive of TB or known HIV infection must provide sputum specimens on 3 consecutive days for acid-fast bacilli smear microscopy, culture (solid and liquid) for *Mycobacterium tuberculosis,* and, if culture-positive, drug susceptibility testing.

**Syphilis and gonorrhea**

During the study period, applicants aged ≥15 years were required to receive testing for syphilis and gonorrhea, and those aged <15 years were required to receive testing if infection was suspected or if they had a history of syphilis or gonorrhea. For syphilis, a nontreponemal serologic test must be used first. Positive or reactive results must be confirmed by a treponemal test. For gonorrhea, beginning October 1, 2016, applicants must receive a nucleic acid amplification test or gonococcal culture; in 2017, the use of culture as a screening option was discontinued. Laboratory testing for gonorrhea was not required before 2016. In 2021, after the study period, the age requirements for testing were narrowed to 18–44 years for syphilis and 18–24 years for gonorrhea. Applicants of any age must still be tested if infection is suspected.

**Hansen’s disease**

All applicants must receive a clinical evaluation for Hansen’s disease. Diagnoses follow the WHO categorization scheme.*

**Source:** CDC. Tuberculosis technical instructions for panel physicians. Atlanta, GA: US Department of Health and Human Services, CDC. https://www.cdc.gov/immigrantrefugeehealth/exams/ti/panel/tuberculosis-panel-technical-instructions.html

* World Health Organization. Classification of leprosy. Geneva, Switzerland: World Health Organization; 2018. https://www.who.int/news-room/fact-sheets/detail/leprosy

#### Overseas Interventions for Refugees

Unlike immigrants, refugees are not required to receive vaccinations for admission to the United States, and many refugees might be undervaccinated when they receive their overseas medical examination, leaving them at risk for vaccine-preventable diseases. Refugees are required to demonstrate documentation of vaccinations when they adjust their status from refugee to immigrant, and they are required to apply for immigrant status after 1 year in the United States. To address this gap, CDC launched a voluntary vaccination program for U.S.-bound refugees in 2012 (*8*). The vaccination program is cofunded by CDC and DOS; the major implementing partner is the International Organization for Migration (IOM). The program provides refugees with the following vaccines, depending on age and eligibility: measles; mumps; rubella; hepatitis B; *Haemophilus influenzae* type B; pneumococcal conjugate vaccine; meningococcal conjugate vaccine with protection against serogroups A, C, W, and Y; diphtheria; tetanus; pertussis; and polio. Refugees typically receive these scheduled or catch-up vaccinations as recommended by the Advisory Committee on Immunization Practices at their initial overseas examination and, when logistically feasible, receive additional doses in each vaccine series before departure for the United States (*8*).

Except when contraindicated, refugees who receive their overseas examination in sub-Saharan Africa are offered albendazole for soil-transmitted helminth infections, ivermectin for *Strongyloides stercoralis* infection in countries where *Loa loa* is not endemic, praziquantel for schistosomiasis, and artemether-lumefantrine for *Plasmodium falciparum* in areas where malaria is endemic. In countries where *L. loa* is endemic, management of *S. stercoralis* is deferred until after arrival in the United States because of the risk for encephalopathy that might be associated with ivermectin treatment when *L. loa* infection is present. Refugees who receive their examinations in the Middle East, Asia, North Africa, Latin America, and the Caribbean are offered albendazole (for soil-transmitted helminths) and ivermectin (for strongyloidiasis) only. Refugees outside these areas, such as European countries and countries formerly in the Union of Soviet Socialist Republics, or those in areas with high infection rates for other parasitic infections (e.g., *Plasmodium vivax*), are offered specific treatment on a case-by-case basis.

#### Postarrival Medical Examination in the United States

CDC recommends a TB examination for immigrants who have class A or B TB according to the overseas examination and recommends a comprehensive assessment that includes a TB examination for all refugees and eligible others (*9*). Appropriate follow-up care in the United States can prevent progression from infection to disease or, if the disease has already developed or recurred since the overseas examination, allow rapid diagnosis and treatment, ultimately limiting spread within the United States. To facilitate outreach for the postarrival examination, CDC notifies state and local health departments whenever such persons arrive in the United States, typically within 1–9 days. U.S. clinicians affiliated with health departments conduct the postarrival examination, typically within 30–90 days. These voluntary follow-up examinations for newly arrived immigrants, refugees, and eligible others should not be confused with the required examination conducted by civil surgeons for adjustment-of-status immigrants who apply for lawful permanent residence status from within the United States.

### Scope and Purpose

This report summarizes health information reported to CDC’s Electronic Disease Notification (EDN) system from overseas medical examinations conducted by panel physicians and postarrival TB examinations overseen by U.S. health departments and describes the number and demographics of immigrants, refugees, and eligible others who arrived in the United States during 2014–2019. The health-related findings from medical examinations conducted overseas and domestically are summarized, with a focus on TB, syphilis, gonorrhea, and Hansen’s disease. Public health interventions among refugees also are described. These findings will help federal, state, and local health departments and other agencies that serve U.S.-bound or recently arrived immigrants, refugees, and eligible others to guide health-related priorities, programming, and policies. 

## Methods

### Data Source and Collection

**FIGURE 1:**
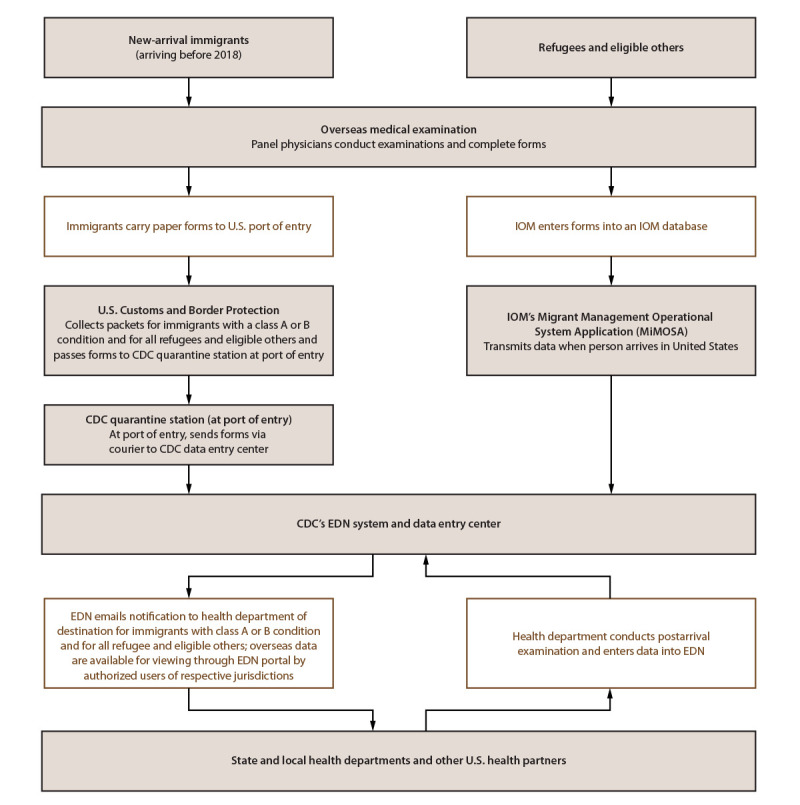
CDC Electronic Disease Notification* system flow chart for new-arrival immigrants, refugees, and eligible others **Abbreviations:** EDN = Electronic Disease Notification; IOM = International Organization for Migration. * Data for immigrants who arrived before 2018 follow the left pathway. In 2018, CDC in collaboration with U.S. Department of State launched the U.S. version of a system called eMedical for processing overseas medical examination data for immigrants. Panel physicians enter data directly into the eMedical system, which are then transferred to the EDN system within 2 days of the immigrant’s arrival in the United States. From 2018 onward, data for most immigrants are transferred to EDN via eMedical. Data for refugees and most eligible others follow the pathway on the right; however, data for certain eligible others follow the left pathway. New-arrival immigrants are persons who, while abroad, completed the application process for lawful permanent residency in the United States. Refugees are persons unable or unwilling to return to their country of nationality because of persecution, or a well-founded fear of persecution, resulting from their race, religion, nationality, membership in a particular social group, or political opinion. Eligible others are persons admitted from abroad, other than refugees, who are eligible for services from the Office of Refugee Resettlement (primarily parolees, Iraqi and Afghan special immigrant visa holders, and follow-to-join asylees). IOM, an intergovernmental organization, has a special role in resettlement. For refugees and many eligible others, IOM organizes safe travel (departure and arrival in the United States) and transmits overseas medical examination data to CDC including, in certain instances, data collected by non-IOM panels.

EDN, which has been described previously (*10*), has both surveillance and programmatic components. The surveillance component is the systematic collection of data from 1) the overseas medical examination as documented by panel physicians for immigrants with class A or B conditions and for all refugees and eligible others and 2) the recommended postarrival TB examination as reported by U.S. health departments for immigrants, refugees, and eligible others with class A or B TB (Figure 1). The programmatic component of EDN is a centralized reporting system. When an immigrant with class A or B TB, a refugee, or an eligible other arrives in the United States, EDN notifies the U.S. health department in the jurisdiction where the newly arriving person reports intending to live. EDN provides a portal for health authorities and other authorized users, such as U.S. clinicians who conduct the recommended follow-up examinations, to access the person’s overseas medical examination record. Health departments can update individual EDN records with results from the postarrival TB examination. This postarrival information is transmitted back to CDC and, if needed, to other health departments when a person moves from one jurisdiction to another.

### Definitions

Immigrants, also known as lawful permanent residents or green card holders, are persons who have been granted lawful permanent residence in the United States (*11*). New-arrival immigrants complete the application process overseas and enter the United States as lawful permanent residents, whereas adjustment-of-status immigrants complete the application process within the United States, having initially entered with a nonimmigrant status and later adjusting to an immigrant status.

Refugees and asylees are persons unable or unwilling to return to their country of nationality because of persecution, or a well-founded fear of persecution, resulting from their race, religion, nationality, membership in a particular social group, or political opinion (*12*,*13*). Parolees are persons granted parole into the United States for humanitarian reasons or substantial public benefit. Refugees and certain parolees complete the admission process overseas. Asylees and other parolees complete the admission process within the United States.

This report includes persons who completed the admission process overseas (new-arrival immigrants, refugees and follow-to-join refugees, and eligible others) and then entered the United States during 2014–2019. The analysis does not include persons who completed the admission process inside the United States (adjustment-of-status immigrants, asylees, and some parolees) and persons who did not complete the admission process (irregular migrants). The population categories used for this analysis included the following:

- **Immigrants:** All new-arrival immigrants except for certain Iraqi and Afghan immigrants (see eligible others category).
- **Refugees:** All refugees, including follow-to-join refugees (eligible relatives of previously admitted refugees, also known as visa 93 holders).
- **Eligible others:** Persons admitted from abroad, other than refugees, who are nonetheless eligible for services from the Office of Refugee Resettlement (ORR), an office of the Administration for Children and Families division of HHS. ORR helps certain populations, primarily those entering the United States through humanitarian programs, with resettlement and local integration. The eligible others category includes parolees and follow-to-join asylees (eligible relatives of previously admitted asylees, also known as visa 92 holders) admitted from abroad. Eligible others also include persons, and their eligible relatives, admitted under special immigrant visa programs for Iraqis and Afghans who served the U.S. government. This category does not include anyone admitted from within the United States, such as asylees, regardless of their eligibility for ORR services.

### Analysis 

EDN data for 2014–2019 were used to describe the number and demographics of newly arrived refugees and eligible others. Because EDN does not collect data for immigrants who do not have a class A or B condition identified overseas, publicly available DHS data were used to determine the number and demographics of immigrants who arrived in the United States during the study period (*14*). EDN also does not collect nationality data. For immigrants, country of birth was used as a proxy for nationality. For refugees, country of birth is a poor proxy for nationality. (Many refugees are born in a host country to refugee parents who fled their home country.) Instead, nationality data extracted from the DOS Worldwide Refugee Admissions Processing System (*15*) were used. For all groups, EDN data were used to determine the number and type of class A or B TB, syphilis, gonorrhea, and Hansen’s disease cases identified overseas (Box 2) and the number of notifications sent to each U.S. state. Because refugees were offered additional, voluntary interventions, including vaccinations and presumptive treatment for parasites, first- and second-dose coverage with measles-containing vaccine among refugees were determined; pregnancy status, contraindications, and other barriers were examined; and the proportion who received presumptive treatment for parasites was calculated.

## Results

**FIGURE 2:**
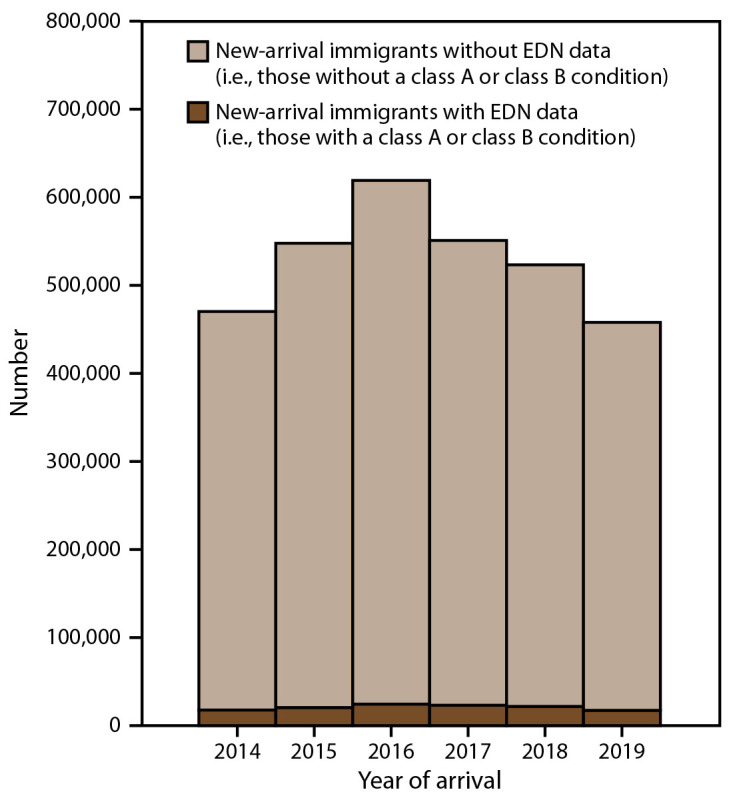
Number of new-arrival immigrants* with and without overseas medical examination data collected by the Electronic Disease Notification system — United States, 2014–2019 **Abbreviation:** EDN = Electronic Disease Notification. * New-arrival immigrants are persons who, while abroad, completed the application process for lawful permanent residency in the United States. Publicly available U.S. Department of Homeland Security data were used to determine the number new-arrival immigrants without EDN data (US Department of Homeland Security. Legal immigration and adjustment of status report quarterly data. Washington, DC: US Department of Homeland Security; 2020. https://www.dhs.gov/immigration-statistics/readingroom/special/LIASR).

**FIGURE 3:**
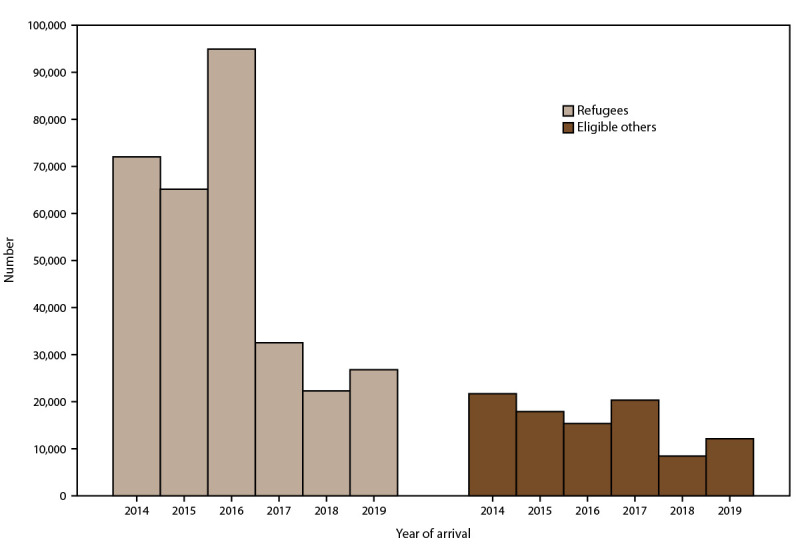
Number of refugees and eligible others* arriving per year — United States, 2014–2019 * Refugees are persons unable or unwilling to return to their country of nationality because of persecution, or a well-founded fear of persecution, resulting from their race, religion, nationality, membership in a particular social group, or political opinion. Eligible others are persons admitted from abroad, other than refugees, who are eligible for services from the Office of Refugee Resettlement (primarily parolees, Iraqi and Afghan special immigrant visa holders, and follow-to-join asylees). The Electronic Disease Notification system collects data for all refugees and eligible others.

During 2014–2019, approximately 3.5 million persons entered the United States as new-arrival immigrants, refugees, or eligible others (Figures 2 and 3). Most (3.2 million) entered as new-arrival immigrants, averaging 528,252 annually (range: 457,930–619,100) (Figure 2). Each year, the largest proportions of immigrants were nationals of Mexico, the Dominican Republic, the People’s Republic of China, the Philippines, Vietnam, India, Bangladesh, El Salvador, Haiti, and Pakistan, and the distribution of nationalities remained relatively constant over time (Supplementary Figure 1, https://stacks.cdc.gov/view/cdc/113063). Among all immigrants, 56% were nationals of these 10 countries. During the same period, 313,890 persons entered as refugees and 95,993 as eligible others (Figure 3). The number of refugees and eligible others who arrived each year declined from an average of 95,715 during 2014–2016 to an average of 40,912 during 2017–2019. Among refugees and eligible others, the largest proportions were nationals of Democratic Republic of Congo, Burma, Iraq, Cuba, Somalia, Bhutan, Syria, Ukraine, Iran, and Eritrea; however, the distribution of nationalities shifted markedly over time (Supplementary Figure 2, https://stacks.cdc.gov/view/cdc/113063).

### Tuberculosis

Among all 3.5 million immigrants, refugees, and eligible others who arrived in the United States during 2014–2019, the overseas examination identified 139,688 (3.9%) persons with class A or B TB, including five persons (0.0001% of entrants) with class A TB admitted with a waiver; 6,586 (0.2% of entrants) with class B0 TB, pulmonary; 94,533 (2.6% of entrants) with class B1 TB, pulmonary; and 38,023, mostly children, with class B2 TB, LTBI evaluation (Table 1). (Because testing for immune reactivity to *M. tuberculosis* is not required for most entrants, this report does not present the proportion of all entrants with class B2, LTBI evaluation.) The proportion of entrants with class A or B TB ranged from 3.7% to 4.1% by year and from 3.7% for immigrants, to 7.1% for refugees, to 1.4% for eligible others.

**TABLE 1:** Characteristics of immigrants, refugees, and eligible others* with a tuberculosis-related classification^†^ identified by the overseas medical examination — United States, 2014–2019

Characteristic	Class A or class B TB	Class A TB with waiver	Class B0 TB, pulmonary	Class B1 TB, pulmonary	Class B1 TB, extrapulmonary	Class B2 TB, LTBI evaluation	Class B3 TB, contact evaluation
	No. (%)	No. (%)	No. (%)	No. (%)	No. (%)	No. (%)	No. (%)
**Total**	**139,688^§^ (100)**	**5 (100)**	**6,586 (100)**	**94,533 (100)**	**394 (100)**	**38,023 (100)**	**2,318 (100)**
**Status at U.S. entry**							
Immigrant	116,180 (83.2)	5 (100)	5,124 (77.8)	79,011 (83.6)	314 (79.7)	31,641 (83.2)	1,806 (77.9)
Refugee	22,256 (15.9)	0 (0)	1,420 (21.6)	14,890 (15.8)	71 (18.0)	5,821 (15.3)	485 (20.9)
Eligible other	1,252 (0.9)	0 (0)	42 (0.6)	632 (0.7)	9 (2.3)	561 (1.5)	27 (1.2)
**Year of U.S. entry**							
2014	21,894 (15.7)	2 (40.0)	1,183 (18.0)	13,219 (14.0)	92 (23.4)	7,443 (19.6)	467 (20.1)
2015	24,157 (17.3)	2 (40.0)	1,175 (17.8)	14,819 (15.7)	83 (21.1)	8,115 (21.3)	396 (17.1)
2016	28,541 (20.4)	1 (20.0)	1,286 (19.5)	18,297 (19.4)	86 (21.8)	8,870 (23.3)	403 (17.4)
2017	23,828 (17.1)	0 (0)	1,106 (16.8)	16,262 (17.2)	58 (14.7)	6,410 (16.9)	329 (14.2)
2018	22,793 (16.3)	0 (0)	980 (14.9)	17,309 (18.3)	36 (9.1)	4,361 (11.5)	411 (17.7)
2019	18,475 (13.2)	0 (0)	856 (13.0)	14,627 (15.5)	39 (9.9)	2,824 (7.4)	312 (13.5)
**Age group (yrs) at overseas examination**							
<2	180 (0.1)	2 (40.0)	16 (0.2)	39 (0.0)	3 (0.8)	113 (0.3)	29 (1.3)
2–14	37,412 (26.8)	3 (60.0)	325 (4.9)	1,185 (1.3)	39 (9.9)	35,814 (94.2)	565 (24.4)
15–24	6,972 (5.0)	0 (0)	816 (12.4)	5,194 (5.5)	51 (12.9)	798 (2.1)	603 (26.0)
25–34	12,806 (9.2)	0 (0)	1,140 (17.3)	11,269 (11.9)	90 (22.8)	339 (0.9)	160 (6.9)
35–44	14,947 (10.7)	0 (0)	1,014 (15.4)	13,535 (14.3)	68 (17.3)	347 (0.9)	196 (8.5)
45–54	18,281 (13.1)	0 (0)	1,037 (15.7)	1,6884 (17.9)	53 (13.5)	290 (0.8)	267 (11.5)
55–64	23,999 (17.2)	0 (0)	1,225 (18.6)	22,463 (23.8)	52 (13.2)	245 (0.6)	314 (13.5)
≥65	25,091 (18.0)	0 (0)	1,013 (15.4)	23,964 (25.3)	38 (9.6)	77 (0.2)	184 (7.9)
**Sex**							
Female	71,616 (51.3)	4 (80.0)	2,899 (44.0)	49,407 (52.3)	214 (54.3)	18,973 (49.9)	1,304 (56.3)
Male	68,072 (48.7)	1 (20.0)	3,687 (56.0)	45,126 (47.7)	180 (45.7)	19,050 (50.1)	1,014 (43.7)
**Country of nationality** ^¶^							
Mexico	12,925 (9.3)	0 (0)	126 (1.9)	7,257 (7.7)	14 (3.6)	5,550 (14.6)	9 (0.4)
Philippines	45,302 (32.4)	0 (0)	2,410 (36.6)	27,674 (29.3)	47 (11.9)	15,108 (39.7)	1,311 (56.6)
India	4,601 (3.3)	1 (20.0)	101 (1.5)	4,224 (4.5)	49 (12.4)	271 (0.7)	5 (0.2)
Vietnam	11,489 (8.2)	0 (0)	1,342 (20.4)	8,266 (8.7)	50 (12.7)	1,857 (4.9)	27 (1.2)
China	7,721 (5.5)	3 (60.0)	360 (5.5)	6,416 (6.8)	15 (3.8)	846 (2.2)	147 (6.3)
Guatemala	388 (0.3)	0 (0)	13 (0.2)	107 (0.1)	3 (0.8)	270 (0.7)	0 (0)
Haiti	1,264 (0.9)	0 (0)	58 (0.9)	744 (0.8)	2 (0.5)	454 (1.2)	15 (0.6)
Honduras	544 (0.4)	0 (0)	5 (0.1)	129 (0.1)	2 (0.5)	409 (1.1)	2 (0.1)
Ethiopia	2,590 (1.9)	0 (0)	36 (0.5)	1,901 (2.0)	18 (4.6)	519 (1.4)	140 (6.0)
Burma	5,846 (4.2)	0 (0)	687 (10.4)	3,886 (4.1)	29 (7.4)	1,248 (3.3)	174 (7.5)
El Salvador	1,376 (1.0)	0 (0)	36 (0.5)	858 (0.9)	0 (0)	477 (1.3)	8 (0.3)
Pakistan	1,003 (0.7)	0 (0)	26 (0.4)	892 (0.9)	9 (2.3)	85 (0.2)	3 (0.1)
Nepal	1,106 (0.8)	0 (0)	46 (0.7)	1,012 (1.1)	4 (1.0)	48 (0.1)	2 (0.1)
Republic of Korea	989 (0.7)	0 (0)	16 (0.2)	850 (0.9)	3 (0.8)	126 (0.3)	1 (0.0)
Somalia	2,880 (2.1)	0 (0)	78 (1.2)	1,845 (2.0)	19 (4.8)	957 (2.5)	10 (0.4)
Nigeria	646 (0.5)	0 (0)	16 (0.2)	498 (0.5)	6 (1.5)	129 (0.3)	4 (0.2)
Cambodia	803 (0.6)	0 (0)	58 (0.9)	699 (0.7)	5 (1.3)	42 (0.1)	6 (0.3)
Peru	862 (0.6)	0 (0)	12 (0.2)	740 (0.8)	0 (0)	108 (0.3)	4 (0.2)
Ecuador	244 (0.2)	0 (0)	3 (0.0)	124 (0.1)	0 (0)	117 (0.3)	0 (0)
Bangladesh	1,416 (1.0)	0 (0)	49 (0.7)	822 (0.9)	7 (1.8)	519 (1.4)	36 (1.6)
Laos	18 (0.0)	0 (0)	3 (0.0)	14 (0.0)	0 (0)	1 (0.0)	0 (0)
Dominican Republic	6,878 (4.9)	0 (0)	90 (1.4)	5,870 (6.2)	8 (2.0)	908 (2.4)	10 (0.4)
Kenya	1,219 (0.9)	0 (0)	32 (0.5)	782 (0.8)	5 (1.3)	400 (1.1)	7 (0.3)
Colombia	1,680 (1.2)	0 (0)	6 (0.1)	976 (1.0)	3 (0.8)	697 (1.8)	1 (0.0)
Bhutan	4,236 (3.0)	0 (0)	432 (6.6)	3,182 (3.4)	16 (4.1)	549 (1.4)	217 (9.4)
Congo	33 (0.0)	0 (0)	0 (0)	19 (0.0)	0 (0)	14 (0.0)	0 (0)
Thailand	361 (0.3)	0 (0)	8 (0.1)	294 (0.3)	2 (0.5)	60 (0.2)	3 (0.1)
Afghanistan	815 (0.6)	0 (0)	27 (0.4)	675 (0.7)	5 (1.3)	112 (0.3)	5 (0.2)
Indonesia	175 (0.1)	0 (0)	13 (0.2)	132 (0.1)	1 (0.3)	27 (0.1)	7 (0.3)
Other	20,278 (14.5)	1 (20.0)	497 (7.5)	13,645 (14.4)	72 (18.3)	6,115 (16.1)	164 (7.1)

**Abbreviations:** LTBI = latent tuberculosis infection; TB = tuberculosis.

* Eligible others are persons admitted from abroad, other than refugees, who are eligible for services from the Office of Refugee Resettlement (primarily parolees, Iraqi and Afghan special immigrant visa holders, and follow-to-join asylees).

^†^ See Box 1 for classification criteria.

^§^ Final TB classifications are mutually exclusive with two exceptions: 1) applicants may be simultaneously categorized as class B1 TB, pulmonary, and class B1 TB, extrapulmonary, and 2) applicants may be simultaneously categorized as class B3 TB, contact evaluation, and any other classifications. Therefore, the number of classifications might be greater than the number of persons with classifications.

^¶^ Nationalities are listed in order of the top 30 birth countries (excluding the United States and its territories) for TB cases reported to the U.S. National TB Surveillance System in 2019.

**FIGURE 4:**
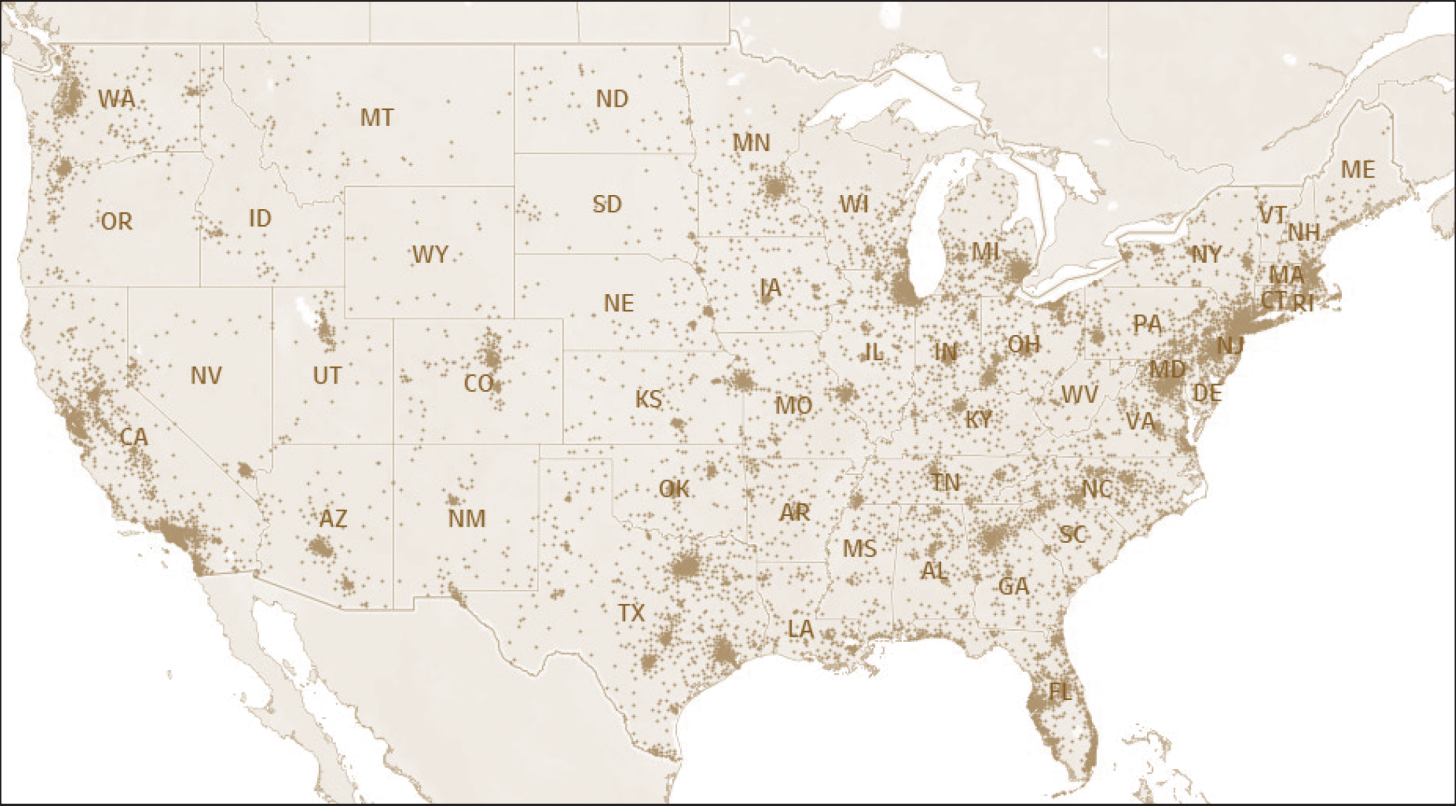
U.S. state and local health departments* that received notifications for arrival of immigrants, refugees, and eligible others^†^ (N = 139,688) with class A or B tuberculosis^§^ — United States, 2014–2019 * U.S. territories and freely associated states are not shown. ^†^ New-arrival immigrants are persons who, while abroad, completed the application process for lawful permanent residency in the United States. Refugees are persons unable or unwilling to return to their country of nationality because of persecution, or a well-founded fear of persecution, resulting from their race, religion, nationality, membership in a particular social group, or political opinion. Eligible others are persons admitted from abroad, other than refugees, who are eligible for services from the Office of Refugee Resettlement (primarily parolees, Iraqi and Afghan special immigrant visa holders, and follow-to-join asylees). ^§^ See Box 1 for classification criteria.

Notifications were sent for all 139,688 persons with class A or B TB to the relevant state or local health agency (Figure 4). This process facilitates a postarrival domestic follow-up examination. California received the most notifications (26.8%), followed by New York (8.7%) and Texas (8.3%). Among persons with class B0 TB, pulmonary, or with class B1 TB, pulmonary, the proportion with a complete postarrival TB examination reported to EDN within 1 year of arrival and ever, respectively, were 65.2% (first year) and 67.0% (ever) for immigrants, 74.8% and 75.7% for refugees, and 52.7% and 54.0% for eligible others (Table 2); the proportion reporting a complete postarrival examination within 1 year of arrival varied by state, with an overall proportion for immigrants, refugees, and eligible others ranging from 23.0% to 92.7% (Table 2). Among children aged 2–14 years with class B2, LTBI evaluation, the proportion with a complete postarrival examination reported to EDN within 1 year of arrival and ever, respectively, were 55.9% (first year) and 58.4% (ever) for immigrants, 70.8% and 72.0% for refugees, and 45.9% and 47.9% for eligible others. The overall proportion for completion within 1 year of arrival by state ranged from 8.8% to 91.4% (Table 3). For all persons with class B TB, the proportion with a complete postarrival TB examination in EDN within 1 year of arrival was lowest in 2019 (Tables 2 and 3).

**TABLE 2:** Results from the postarrival tuberculosis examinations in the United States among immigrants, refugees, and eligible others* identified overseas with class B0 or B1 tuberculosis, pulmonary^†^ — United States, 2014–2019

Characteristic	Class B0 or B1 TB	Completed U.S. examination^§^ (% of class B0 or B1 TB)	Diagnosis from U.S. examination (% of completed examinations)			
			TB disease			LTBI
			Culture positive	Culture negative	Culture result not reported	
	No.	No. (%)	No. (%)	No. (%)	No. (%)	No. (%)
**Total**	**101,119**	**67,432 (66.7)**	**464 (0.7)**	**460 (0.7)**	**83 (0.1)**	**18,392 (27.3)**
**Overseas TB classification** ^§^						
Class B0 TB, pulmonary	6,586	4,641 (70.5)	6 (0.1)	27 (0.6)	11 (0.2)	338 (7.3)
Class B1 TB, pulmonary	94,533	62,791 (66.4)	458 (0.7)	433 (0.7)	72 (0.1)	18,054 (28.8)
**Status at U.S. entry**						
Immigrant	84,135	54,882 (65.2)	413 (0.8)	326 (0.6)	56 (0.1)	13,928 (25.4)
Refugee	16,310	12,195 (74.8)	47 (0.4)	126 (1.0)	25 (0.2)	4,364 (35.8)
Eligible other	674	355 (52.7)	4 (1.1)	8 (2.3)	2 (0.6)	100 (28.2)
**Year of U.S. entry**						
2014	14,402	9,300 (64.6)	66 (0.7)	75 (0.8)	21 (0.2)	2,807 (30.2)
2015	15,994	9,996 (62.5)	69 (0.7)	82 (0.8)	16 (0.2)	2,760 (27.6)
2016	19,583	13,971 (71.3)	91 (0.7)	101 (0.7)	10 (0.1)	3,777 (27.0)
2017	17,368	12,019 (69.2)	91 (0.8)	76 (0.6)	15 (0.1)	3,178 (26.4)
2018	18,289	12,704 (69.5)	89 (0.7)	73 (0.6)	12 (0.1)	3,502 (27.6)
2019	15,483	9,442 (61.0)	58 (0.6)	53 (0.6)	9 (0.1)	2,368 (25.1)
**Age group (yrs) at overseas examination**						
<2	55	28 (50.9)	0 (0)	0 (0)	2 (7.1)	6 (21.4)
2–14	1,510	1,032 (68.3)	7 (0.7)	12 (1.2)	8 (0.8)	323 (31.3)
15–24	6,010	4,064 (67.6)	54 (1.3)	59 (1.5)	6 (0.1)	978 (24.1)
25–34	12,409	8,392 (67.6)	85 (1.0)	69 (0.8)	17 (0.2)	2,308 (27.5)
35–44	14,549	9,862 (67.8)	59 (0.6)	68 (0.7)	14 (0.1)	2,778 (28.2)
45–54	17,921	12,005 (67.0)	66 (0.5)	71 (0.6)	11 (0.1)	3,437 (28.6)
55–64	23,688	15,689 (66.2)	93 (0.6)	91 (0.6)	14 (0.1)	4,316 (27.5)
≥65	24,977	16,360 (65.5)	100 (0.6)	90 (0.6)	11 (0.1)	4,246 (26.0)
**Sex**						
Female	52,306	34,860 (66.6)	206 (0.6)	205 (0.6)	46 (0.1)	9,292 (26.7)
Male	48,813	32,572 (66.7)	258 (0.8)	255 (0.8)	37 (0.1)	9,100 (27.9)
**U.S. destination state/area** ^¶^						
Alabama	312	213 (68.3)	3 (1.4)	1 (0.5)	1 (0.5)	64 (30.0)
Alaska	472	373 (79.0)	5 (1.3)	2 (0.5)	0 (0)	152 (40.8)
Arizona	1,746	1,175 (67.3)	9 (0.8)	9 (0.8)	0 (0)	431 (36.7)
Arkansas	236	143 (60.6)	0 (0)	0 (0)	1 (0.7)	51 (35.7)
California	26,849	17,013 (63.4)	154 (0.9)	72 (0.4)	8 (0.0)	3,364 (19.8)
Colorado	1,263	1,033 (81.8)	6 (0.6)	5 (0.5)	1 (0.1)	266 (25.8)
Connecticut	684	213 (31.1)	2 (0.9)	0 (0)	2 (0.9)	61 (28.6)
Delaware	138	69 (50.0)	0 (0)	0 (0)	0 (0)	23 (33.3)
District of Columbia	174	40 (23.0)	0 (0)	1 (2.5)	0 (0)	11 (27.5)
Florida	4,118	2,851 (69.2)	18 (0.6)	51 (1.8)	1 (0.0)	806 (28.3)
Georgia	2,338	1,523 (65.1)	5 (0.3)	9 (0.6)	3 (0.2)	582 (38.2)
Hawaii	2,716	2,464 (90.7)	26 (1.1)	28 (1.1)	1 (0.0)	635 (25.8)
Idaho	322	230 (71.4)	1 (0.4)	2 (0.9)	3 (1.3)	96 (41.7)
Illinois	4,068	2,991 (73.5)	14 (0.5)	42 (1.4)	8 (0.3)	746 (24.9)
Indiana	1,123	824 (73.4)	8 (1.0)	16 (1.9)	2 (0.2)	342 (41.5)
Iowa	650	500 (76.9)	2 (0.4)	6 (1.2)	2 (0.4)	178 (35.6)
Kansas	553	128 (23.1)	1 (0.8)	0 (0)	0 (0)	50 (39.1)
Kentucky	824	570 (69.2)	2 (0.4)	8 (1.4)	1 (0.2)	213 (37.4)
Louisiana	378	211 (55.8)	1 (0.5)	2 (0.9)	1 (0.5)	55 (26.1)
Maine	145	46 (31.7)	0 (0)	1 (2.2)	0 (0)	26 (56.5)
Maryland	1,981	1,334 (67.3)	11 (0.8)	9 (0.7)	2 (0.1)	406 (30.4)
Massachusetts	1,317	924 (70.2)	6 (0.6)	8 (0.9)	3 (0.3)	334 (36.1)
Michigan	1,695	1,245 (73.5)	7 (0.6)	5 (0.4)	1 (0.1)	361 (29.0)
Minnesota	2,014	1,448 (71.9)	15 (1.0)	8 (0.6)	5 (0.3)	577 (39.8)
Mississippi	171	120 (70.2)	1 (0.8)	1 (0.8)	0 (0)	40 (33.3)
Missouri	819	198 (24.2)	0 (0)	0 (0)	0 (0)	76 (38.4)
Montana	69	19 (27.5)	0 (0)	0 (0)	0 (0)	9 (47.4)
Nebraska	659	257 (39.0)	0 (0)	0 (0)	0 (0)	74 (28.8)
Nevada	1,822	1,609 (88.3)	16 (1.0)	5 (0.3)	1 (0.1)	595 (37.0)
New Hampshire	199	58 (29.1)	0 (0)	1 (1.7)	0 (0)	16 (27.6)
New Jersey	4,044	2,182 (54.0)	6 (0.3)	8 (0.4)	5 (0.2)	607 (27.8)
New Mexico	310	205 (66.1)	1 (0.5)	0 (0)	0 (0)	65 (31.7)
New York**	9,531	6,337 (66.5)	40 (0.6)	36 (0.6)	2 (0.0)	1,507 (23.8)
North Carolina	1,724	1,369 (79.4)	1 (0.1)	5 (0.4)	2 (0.1)	489 (35.7)
North Dakota	289	214 (74.0)	1 (0.5)	1 (0.5)	0 (0)	99 (46.3)
Ohio	2,296	1,158 (50.4)	2 (0.2)	14 (1.2)	1 (0.1)	346 (29.9)
Oklahoma	559	468 (83.7)	0 (0)	1 (0.2)	1 (0.2)	107 (22.9)
Oregon	1,041	877 (84.2)	2 (0.2)	6 (0.7)	1 (0.1)	238 (27.1)
Pennsylvania	2,704	1,869 (69.1)	12 (0.6)	12 (0.6)	5 (0.3)	655 (35.0)
Rhode Island	172	109 (63.4)	1 (0.9)	0 (0)	0 (0)	33 (30.3)
South Carolina	438	339 (77.4)	1 (0.3)	0 (0)	1 (0.3)	128 (37.8)
South Dakota	233	216 (92.7)	1 (0.5)	0 (0)	1 (0.5)	100 (46.3)
Tennessee	1,026	871 (84.9)	6 (0.7)	10 (1.1)	1 (0.1)	269 (30.9)
Texas	7,995	5,049 (63.2)	37 (0.7)	44 (0.9)	9 (0.2)	1,267 (25.1)
Utah	583	527 (90.4)	3 (0.6)	2 (0.4)	0 (0)	193 (36.6)
Vermont	162	111 (68.5)	0 (0)	0 (0)	0 (0)	42 (37.8)
Virginia	2,207	1,712 (77.6)	27 (1.6)	14 (0.8)	1 (0.1)	441 (25.8)
Washington	3,948	3,011 (76.3)	9 (0.3)	9 (0.3)	5 (0.2)	845 (28.1)
West Virginia	81	46 (56.8)	0 (0)	0 (0)	0 (0)	15 (32.6)
Wisconsin	834	603 (72.3)	1 (0.2)	6 (1.0)	1 (0.2)	194 (32.2)
Wyoming	40	23 (57.5)	0 (0)	0 (0)	0 (0)	3 (13.0)

**Abbreviations:** LTBI = latent tuberculosis infection; TB = tuberculosis.

* Eligible others are persons admitted from abroad, other than refugees, who are eligible for services from the Office of Refugee Resettlement (primarily parolees, Iraqi and Afghan special immigrant visa holders, and follow-to-join asylees).

^†^ See Box 1 for classification criteria.

^§^ Persons who completed a U.S. examination within the first year of arrival.

^¶^ U.S. territories and freely associated states are not shown.

** In 2014 and 2015, notifications were substantially delayed for newly arriving immigrants with class B TB who reported intending to live in New York. These delays might have resulted in losses to follow-up and decreased the proportion of persons who completed a U.S. examination in this state.

**TABLE 3:** Results from postarrival tuberculosis examinations in the United States among immigrants, refugees, and eligible others* aged 2–14 years identified overseas with class B2 tuberculosis, latent tuberculosis infection evaluation^†^ — United States, 2014–2019

Characteristic	Children aged 2–14 yrs with class B2 TB, LTBI evaluation	Completed U.S. examination**^§^** (% of class B2)	Diagnosis from U.S. examination (% of completed examinations)			
			TB disease			LTBI
			Culture positive	Culture negative	Culture result not reported	
	No.	No. (%)	No. (%)	No. (%)	No. (%)	No. (%)
**Total**	**35,814**	**20,758 (58.0)**	**11 (0.1)**	**21 (0.1)**	**16 (0.1)**	**10,223 (49.3)**
**Status at U.S. entry**						
Immigrant	29,887	16,700 (55.9)	4 (0.0)	12 (0.1)	10 (0.1)	8,013 (48.0)
Refugee	5,380	3,807 (70.8)	7 (0.2)	9 (0.2)	6 (0.2)	2,083 (54.7)
Eligible other	547	251 (45.9)	0 (0.0)	0 (0.0)	0 (0.0)	127 (50.6)
**Year of U.S. entry**						
2014	6,939	4,059 (58.5)	3 (0.1)	8 (0.2)	9 (0.2)	2,141 (52.7)
2015	7,794	4,418 (56.7)	3 (0.1)	5 (0.1)	0 (0.0)	2,213 (50.1)
2016	8,549	5,112 (59.8)	2 (0.0)	2 (0.0)	3 (0.1)	2,440 (47.7)
2017	6,130	3,510 (57.3)	3 (0.1)	1 (0.0)	1 (0.0)	1,514 (43.1)
2018	4,010	2,351 (58.6)	0 (0.0)	1 (0.0)	2 (0.1)	1,100 (46.8)
2019	2,392	1,308 (54.7)	0 (0.0)	4 (0.3)	1 (0.1)	815 (62.3)
**Sex**						
Female	17,742	10,184 (57.4)	6 (0.1)	12 (0.1)	9 (0.1)	4,993 (49.0)
Male	18,072	10,574 (58.5)	5 (0.0)	9 (0.1)	7 (0.1)	5,230 (49.5)
**U.S. destination state/area^¶^**						
Alabama	128	41 (32.0)	0 (0.0)	0 (0.0)	0 (0.0)	13 (31.7)
Alaska	305	245 (80.3)	0 (0.0)	0 (0.0)	0 (0.0)	112 (45.7)
Arizona	662	417 (63.0)	0 (0.0)	1 (0.2)	0 (0.0)	214 (51.3)
Arkansas	119	67 (56.3)	0 (0.0)	0 (0.0)	1 (1.5)	26 (38.8)
California	9,845	3,803 (38.6)	0 (0.0)	3 (0.1)	2 (0.1)	1,479 (38.9)
Colorado	405	325 (80.2)	0 (0.0)	0 (0.0)	0 (0.0)	138 (42.5)
Connecticut	215	60 (27.9)	0 (0.0)	0 (0.0)	0 (0.0)	31 (51.7)
Delaware	44	16 (36.4)	0 (0.0)	0 (0.0)	0 (0.0)	2 (12.5)
District of Columbia	77	24 (31.2)	0 (0.0)	0 (0.0)	0 (0.0)	7 (29.2)
Florida	1,652	1,095 (66.3)	0 (0.0)	2 (0.2)	2 (0.2)	419 (38.3)
Georgia	791	511 (64.6)	0 (0.0)	1 (0.2)	0 (0.0)	285 (55.8)
Hawaii	1,420	1,298 (91.4)	0 (0.0)	0 (0.0)	0 (0.0)	393 (30.3)
Idaho	171	120 (70.2)	0 (0.0)	1 (0.8)	0 (0.0)	58 (48.3)
Illinois	1,500	1,019 (67.9)	1 (0.1)	0 (0.0)	0 (0.0)	380 (37.3)
Indiana	396	276 (69.7)	0 (0.0)	0 (0.0)	0 (0.0)	192 (69.6)
Iowa	181	146 (80.7)	0 (0.0)	0 (0.0)	0 (0.0)	85 (58.2)
Kansas	200	41 (20.5)	0 (0.0)	0 (0.0)	1 (2.4)	18 (43.9)
Kentucky	312	191 (61.2)	0 (0.0)	2 (1.0)	0 (0.0)	105 (55.0)
Louisiana	152	98 (64.5)	0 (0.0)	0 (0.0)	0 (0.0)	40 (40.8)
Maine	43	8 (18.6)	0 (0.0)	0 (0.0)	0 (0.0)	7 (87.5)
Maryland	598	402 (67.2)	0 (0.0)	0 (0.0)	0 (0.0)	199 (49.5)
Massachusetts	340	239 (70.3)	0 (0.0)	2 (0.8)	0 (0.0)	156 (65.3)
Michigan	545	420 (77.1)	2 (0.5)	0 (0.0)	1 (0.2)	144 (34.3)
Minnesota	625	448 (71.7)	2 (0.4)	1 (0.2)	1 (0.2)	219 (48.9)
Mississippi	80	62 (77.5)	0 (0.0)	0 (0.0)	0 (0.0)	16 (25.8)
Missouri	356	80 (22.5)	0 (0.0)	0 (0.0)	0 (0.0)	60 (75.0)
Montana	49	11 (22.4)	0 (0.0)	0 (0.0)	0 (0.0)	4 (36.4)
Nebraska	187	74 (39.6)	0 (0.0)	0 (0.0)	0 (0.0)	33 (44.6)
Nevada	758	676 (89.2)	0 (0.0)	2 (0.3)	2 (0.3)	564 (83.4)
New Hampshire	57	5 (8.8)	0 (0.0)	0 (0.0)	0 (0.0)	3 (60.0)
New Jersey	1,101	634 (57.6)	0 (0.0)	0 (0.0)	0 (0.0)	235 (37.1)
New Mexico	120	92 (76.7)	1 (1.1)	0 (0.0)	0 (0.0)	38 (41.3)
New York**	2,398	1,475 (61.5)	1 (0.1)	2 (0.1)	0 (0.0)	994 (67.4)
North Carolina	614	504 (82.1)	0 (0.0)	0 (0.0)	0 (0.0)	413 (81.9)
North Dakota	113	64 (56.6)	0 (0.0)	0 (0.0)	0 (0.0)	31 (48.4)
Ohio	741	276 (37.2)	0 (0.0)	0 (0.0)	0 (0.0)	140 (50.7)
Oklahoma	220	181 (82.3)	0 (0.0)	0 (0.0)	1 (0.6)	76 (42.0)
Oregon	408	353 (86.5)	1 (0.3)	0 (0.0)	1 (0.3)	175 (49.6)
Pennsylvania	714	440 (61.6)	0 (0.0)	1 (0.2)	1 (0.2)	262 (59.5)
Rhode Island	50	38 (76.0)	0 (0.0)	0 (0.0)	0 (0.0)	17 (44.7)
South Carolina	158	124 (78.5)	0 (0.0)	0 (0.0)	0 (0.0)	37 (29.8)
South Dakota	86	72 (83.7)	0 (0.0)	0 (0.0)	0 (0.0)	48 (66.7)
Tennessee	393	313 (79.6)	0 (0.0)	0 (0.0)	0 (0.0)	156 (49.8)
Texas	3,326	1,853 (55.7)	1 (0.1)	1 (0.1)	2 (0.1)	927 (50.0)
Utah	214	158 (73.8)	0 (0.0)	1 (0.6)	0 (0.0)	111 (70.3)
Vermont	33	16 (48.5)	0 (0.0)	0 (0.0)	0 (0.0)	12 (75.0)
Virginia	709	540 (76.2)	1 (0.2)	1 (0.2)	0 (0.0)	296 (54.8)
Washington	1,450	1,111 (76.6)	1 (0.1)	0 (0.0)	1 (0.1)	704 (63.4)
West Virginia	21	18 (85.7)	0 (0.0)	0 (0.0)	0 (0.0)	5 (27.8)
Wisconsin	270	212 (78.5)	0 (0.0)	0 (0.0)	0 (0.0)	98 (46.2)
Wyoming	24	15 (62.5)	0 (0.0)	0 (0.0)	0 (0.0)	5 (33.3)

**Abbreviations:** LTBI = latent tuberculosis infection; TB = tuberculosis.

* Eligible others are persons admitted from abroad, other than refugees, who are eligible for services from the Office of Refugee Resettlement (primarily parolees, Iraqi and Afghan special immigrant visa holders, and follow-to-join asylees).

^†^ See Box 1 for classification criteria.

^§^ Persons who completed a U.S. examination within the first year of arrival.

^¶^ U.S. territories and freely associated states are not shown.

** In 2014 and 2015, notifications were substantially delayed for newly arriving immigrants with class B TB who reported intending to live in New York. These delays might have resulted in losses to follow-up and decreased the proportion of persons who completed a U.S. examination in this state.

Among persons with a complete postarrival examination, culture-positive TB was diagnosed domestically in six persons (0.1%) identified overseas with class B0 TB, pulmonary, and for 458 (0.7%) persons identified with class B1 TB, pulmonary; among both groups together, the proportion with culture-positive TB diagnosed domestically remained constant over time, ranging from 0.6% to 0.8% during 2014–2019 (Table 2). Among children who were identified overseas with class B2, LTBI evaluation, and had a complete postarrival examination, culture-positive TB was diagnosed domestically among 11 children (0.05%), and LTBI was diagnosed among 10,223 (49.3%) (Table 3).

### Syphilis, Gonorrhea, and Hansen’s Disease

During the study period, overseas evaluations for syphilis and gonorrhea were required for all persons aged ≥15 years. For children aged <15 years, evaluations were required when infection was suspected or a child had a history of infection. For syphilis, overseas laboratory test results were recorded for 94.9% of the 260,345 refugees and eligible others aged ≥15 years who arrived during 2014–2019. The proportion with results increased over time, reaching >99% among those examined in 2018–2019. A total of 1,025 syphilis cases were identified, a rate of 414.9 per 100,000 persons with recorded test results. A reporting requirement for syphilis stage was introduced in 2014. For refugees and eligible others who arrived after 2014, 54 primary or secondary syphilis cases (29.5 per 100,000 persons with test results) and 761 latent syphilis cases (415.3 per 100,000 persons with test results) were identified overseas; the latter included 248 cases of unknown duration. Rates for primary and secondary syphilis were highest among persons aged ≥30 years, and rates for latent syphilis increased with each age group (Table 4). For gonorrhea, a requirement for laboratory testing was introduced in 2016, and reliable data are only available for those examined in 2018 or later. Among 35,653 refugees and eligible others aged ≥15 years examined in 2018–2019, a gonorrhea test result was documented for 98.3%; 131 cases of gonorrhea were identified (373.7 per 100,000 persons with test results), and rates were highest among persons aged 15–34 years. Persons of all ages are screened for Hansen’s disease during their overseas medical examination; among all 409,883 refugees and eligible others who arrived during 2014–2019, a total of 25 had a diagnosis of Hansen’s disease (6.1 per 100,000 persons examined).

**TABLE 4:** Number and rate of refugees and eligible others* with syphilis,^†^ gonorrhea,^§^ or Hansen’s disease identified by the overseas medical examination — United States, 2014–2019

Characteristic	Syphilis (ages ≥15 yrs)						Gonorrhea (ages ≥15 years)		Hansen’s disease (all ages)	
	Primary or secondary		Latent or unknown duration		Total^¶^					
	No.	Rate**	No.	Rate**	No.	Rate**	No.	Rate^††^	No.	Rate
**Total**	**54**	**29.5**	**761** ^§§^	**415.3**	**1,025**	**414.9**	**131**	**373.7**	**25**	**6.1**
**Status at U.S. entry**										
Refugee	52	35.1	689	465.7	**746**	**504.2**	119	454.9	23	7.3
Other	2	5.7	72	204.1	**74**	**209.7**	12	134.9	2	2.1
**Sex**										
Female	25	27.7	328	363.2	**353**	**390.9**	77	443.9	15	7.5
Male	29	31.2	433	466.0	**467**	**502.6**	54	304.9	10	4.8
**Year of arrival**										
2014	—^†^	—^†^	—^†^	—^†^	**205**	**321.3**	—^§^	—^§^	4	4.3
2015	24	46.0	193	370.1	**217**	**416.1**	—^§^	—^§^	9	10.8
2016	17	28.3	225	374.7	**245**	**408.0**	—^§^	—^§^	8	7.3
2017	8	26.7	109	364.0	**117**	**390.7**	—^§^	—^§^	3	5.7
2018	4	21.9	114	625.0	**118**	**647.0**	58	470.6	0	0
2019	1	4.4	120	524.9	**123**	**538.1**	73	321.1	1	2.6
**Age group (yrs) at overseas examination**										
<2	—^¶¶^	—^¶¶^	—^¶¶^	—^¶¶^	**—^¶¶^**	**—^¶¶^**	—^¶¶^	—^¶¶^	0	0
2–14	—^¶¶^	—^¶¶^	—^¶¶^	—^¶¶^	**—^¶¶^**	**—^¶¶^**	—^¶¶^	—^¶¶^	2	1.6
15–19	2	6.7	32	107.5	**35**	**117.6**	17	289.0	1	2.5
20–24	5	17.7	50	176.8	**55**	**194.5**	38	667.5	4	10.2
25–29	6	20.2	80	269.4	**87**	**293**	25	429.6	1	2.4
30–34	9	33.1	77	283.2	**87**	**320**	28	514.2	2	5.4
35–39	10	49.5	100	495.2	**110**	**544.7**	9	234.1	1	3.6
40–44	5	34.1	89	607.0	**95**	**647.9**	4	163.1	2	9.0
45–54	11	59.5	169	913.9	**180**	**973.4**	7	224.3	6	21.1
55–64	3	32.3	101	1086.7	**105**	**1129.8**	3	172.5	4	29.3
≥65	3	52.8	63	1109.5	**66**	**1162.4**	0	0	2	23.6
**Country of nationality**										
Afghanistan	0	0	37	169.3	**37**	**169.3**	19	265.9	1	2.1
Bhutan	12	115.8	4	38.6	**16**	**154.4**	1	434.8	5	22.4
Burma	3	11.9	84	332.1	**87**	**344.0**	6	142.8	5	8.9
Democratic Republic of Congo	17	58.4	292	1003.2	**313**	**1,075.4**	87	860.7	3	5.1
Cuba	2	24.8	12	148.7	**15**	**185.8**	0	0	2	5.6
Iran	0	0	7	95.6	**7**	**95.6**	0	0	0	0
Iraq	2	10.8	56	302.0	**58**	**312.8**	2	282.5	1	1.9
Somalia	2	16.4	71	581.9	**73**	**598.3**	0	0	1	3.2
Syria	0	0	6	56.3	**6**	**56.3**	0	0	1	4.6
Ukraine	0	0	3	30.0	**3**	**30.0**	0	0	1	6.2
Other	16	53.6	189	633.7	**205**	**687.3**	16	209.8	5	8.7

* Eligible others are persons admitted from abroad, other than refugees, who are eligible for services from the Office of Refugee Resettlement (primarily parolees, Iraqi and Afghan special immigrant visa holders, and follow-to-join asylees).

^†^ Requirement for reporting syphilis stage was introduced in 2014. For persons who arrived before 2015, data on syphilis stage are incomplete, and this group is excluded from counts and rate calculations for primary, secondary, and latent syphilis but included in counts and rate calculations for all syphilis.

^§^ Requirement for laboratory testing for gonorrhea was introduced in 2016. For persons who were examined before 2018, data for laboratory-confirmed gonorrhea infection are incomplete, and this group is excluded from counts and rate calculations for gonorrhea.

^¶^ Includes 48 syphilis cases staged as primary, six as secondary, 761 as latent or missing, 205 as unstaged in 2014, four as tertiary, and one as neurosyphilis.

** Per 100,000 persons. Syphilis testing was not documented for 5.1% of persons aged ≥15 years. Persons not tested are excluded from all rate calculations.

^††^ Per 100,000 persons. Gonorrhea testing was not documented for 1.7% of persons aged ≥15 years examined during 2018–2019. Persons not tested are excluded from all rate calculations.

^§§^ Includes 513 syphilis cases staged as latent and 248 of unknown duration.

^¶¶^ Testing for syphilis and gonorrhea are not required for children aged <15 years unless infection is suspected or the child has a history of syphilis or gonorrhea.

### Vaccination Program and Presumptive Treatment for Refugees

CDC’s vaccination program for refugees began in December 2012 in two countries, Thailand and Nepal. By 2014, the program operated in 39 of 89 countries where overseas medical examinations for refugees were performed. Among all refugees who arrived that year, EDN data showed that first- and second-dose coverage with measles-containing vaccine for 66,727 eligible refugees (those born after 1956, who were aged at least 1 year before departing for the United States, and titers, if available, do not indicate immunity) were 49% and 41%, respectively. By 2019, the vaccination program had expanded to all 73 countries where examinations for 22,142 refugees were performed and for that calendar year achieved first-dose coverage of 96% and second-dose coverage of 80%. Among 846 eligible refugees who did not receive a first dose overseas, 17% were pregnant, 15% had another contraindication, 31% were subject to local vaccine shortages, and 6% did not have enough time to get vaccinated. Live virus vaccines, such as measles vaccine, are not routinely administered within 28 days of departure for the United States to avoid interference with any live vaccines or tests of immune response for *M. tuberculosis* antigen testing administered shortly after arrival in the United States (*16*).

CDC’s presumptive treatment program for soil-transmitted helminthiasis, strongyloidiasis, schistosomiasis, and malaria infection varies by country. In populations for whom treatment was recommended, the proportion with documented treatment was 96% for albendazole, 79% for ivermectin (increasing to 84% when countries in which ivermectin is not licensed were excluded), 87% for praziquantel, and 93% for artemether-lumefantrine.

## Discussion

During 2014–2019, approximately 3.5 million persons moved to the United States as an immigrant, a refugee, or an eligible other. Among immigrants, the number of arrivals and distribution of nationalities changed little over time. Among refugees and eligible others, both the number of arrivals and distribution of nationalities changed substantially from year to year; this variability follows U.S. policy decisions. The maximum number of refugees admitted for resettlement is determined by the U.S. president each year. Historically, the actual number of arrivals is equal to or just below that maximum, with some recent exceptions. For fiscal year 2017, the maximum was initially set at 110,000 by President Barack Obama (*17*) and was then decreased to 50,000 by a presidential executive order issued by President Donald Trump (*18*); the actual number of resettled refugees totaled 53,716 for that year (*15*). In fiscal year 2018, although the maximum was set at 45,000 (*19*), approximately one half this number of refugees were resettled after security enhancements were enacted by DHS, resulting in the lowest number of refugee admissions since fiscal year 1977 (*15*). Among eligible others, such as parolees or special immigrant visa holders, numbers and nationalities increase and decrease as programs start and stop. For example, the ending in 2017 of the so-called “wet-foot/dry-foot” policy (which allowed Cuban migrants who arrived in the United States [“dry foot”] without a visa to pursue residency a year later, whereas those intercepted at sea [“wet foot”] were returned to Cuba or resettled in a third country) (*20*) is reflected in the sharp decrease in the number of Cuban parolees in subsequent years. Because health profiles of refugees and eligible others differ by country of origin, country of residence, and local conditions before, during, and after leaving their home country, substantial changes in number and nationality can result in considerable changes in health and public health needs from year to year, posing significant operational challenges for optimizing, or even just preserving, public health programs and infrastructure for these populations, especially at the local level. CDC provides health profiles for each of the largest refugee groups resettling to the United States that describe the demographic, cultural, and health characteristics for each population and are intended to provide clinicians with knowledge needed to better serve these refugees (*21*).

Statutes to exclude non–U.S.-born persons from admission to the United States have been in place for more than a century. These statutes were codified to exclude those with “tuberculosis in any form, or with leprosy, or any contagious disease” in the Immigration and Nationality Act of 1952 and were recodified to exclude those with “a communicable disease of public health significance” (*4*) in accordance with regulations prescribed by the Secretary of HHS in the Immigration of Act of 1990. In 1991, the definition of a communicable disease of public health significance was limited to eight specified conditions: chancroid, gonorrhea, granuloma inguinale, HIV infection, infectious Hansen’s disease, lymphogranuloma venereum, infectious syphilis, and infectious TB (*22*). To allow for a more flexible, risk-based, and responsive approach grounded by medical and epidemiologic factors, this list was expanded in 2009 to include quarantinable communicable diseases, which are designated by a presidential executive order, and communicable PHEIC diseases that could be imported into the United States and affect U.S. residents (*23*). Finally, the list of eight specified conditions was reduced to four with the removal of HIV infection in 2010 (*24*), followed by the removal of chancroid, granuloma inguinale, and lymphogranuloma venereum in 2016 (*25*), leaving only infectious TB, infectious syphilis, gonorrhea, and infectious Hansen’s disease as the remaining specified conditions and the subjects of this report.

### Tuberculosis

Eliminating TB in the United States, defined by the Advisory Committee for Elimination of Tuberculosis as a case rate of <1 per 1 million population, remains a major public health objective (*26*). Despite a historic low in U.S. TB incidence in 2019 of 2.7 cases per 100,000 population, the rate of decline has slowed in recent years (*27*). Persons not born in the United States account for more than two thirds of U.S. cases (*27*), and this proportion is increasing. Genotype studies suggest that most U.S. TB cases in non–U.S.-born persons are due to reactivation of LTBI, likely acquired abroad (*28*,*29*). CDC’s technical instructions are designed to 1) prevent importation of the disease by detecting and treating infectious TB disease (class A TB) before persons move to the United States and 2) reduce community transmission in the general U.S. population by identifying persons with conditions associated with increased risk for disease (class B TB) so that they can quickly receive testing and, if needed, preventive or curative treatment after moving.

EDN data are critical for monitoring implementation of the technical instructions and for evaluating the impact for continuous improvement. Before 2007, U.S.-bound immigrants, refugees, and eligible others were screened for TB by an algorithm based on sputum smear results. This algorithm was unable to identify persons entering the United States with TB disease with negative sputum smears but positive cultures, and a large proportion (one half to two thirds) of cases were likely being missed (*30*,*31*). To address this gap, CDC updated the technical instructions to require culture in addition to sputum smears and, for those with positive smear or culture test results, completion of a course of treatment administered by direct observation supervised by a panel physician. Started in 2007, full global implementation was achieved in 2013. A previous analysis of EDN data found that the annual number of smear-negative but culture-positive TB cases diagnosed during the overseas medical examination increased monotonically from four cases in 2007 to 629 in 2012, whereas annual TB cases detected among non–U.S.**-**born persons within the first year of arrival in the United States decreased from 1,511 to 940 cases, concurrent with overall decreases in the U.S. TB rates (*27*,*30*). In other words, by 2012, approximately 600 more cases were detected overseas at the same time 600 fewer were detected in the United States. This finding suggests that the culture-based algorithm detects substantially more cases, resulting in subsequent treatment, during the overseas medical examination process, thus reducing importation. However, the findings in this report indicate that during 2014–2019, among persons with an overseas classification of class B0 TB, pulmonary, or class B1 TB, pulmonary, the proportion with a subsequent postarrival diagnosis of culture-positive TB disease during their U.S. TB examination remained steady over time, suggesting that new gains will require new strategies. Finally, during this same period, the proportion of persons with class B TB for whom a postarrival TB examination was reported to EDN as completed was lowest in 2019, a particularly concerning finding because of the subsequent spread of SARS-CoV-2, the virus that causes COVID-19, which diverted focus and resources away from TB control and other routine public health measures at the local, state, and federal levels beginning in 2020 (*32*).

In October 2018, updates to the technical instructions introduced refinements to the TB classification schema for the overseas examination. After the update, persons with an initial diagnosis of TB disease who subsequently completed directly observed therapy under the supervision of a panel physician (class B0 TB, pulmonary) are distinguished from those who, during their initial examination, had signs, symptoms, or chest radiography suggestive of TB but negative results from smear and culture tests (class B1 TB, pulmonary). Before the change, the technical instructions aggregated both groups together (previously class B1 TB, pulmonary, for both). This report and others (*33*) show that persons who meet current criteria for class B1 TB, pulmonary, are more likely to receive a diagnosis of culture-positive TB disease at their postarrival follow-up examination than those meeting criteria for class B0 TB, pulmonary. Changes to the classification schema to reflect this risk difference might help U.S. health departments better stratify risk for improved patient management.

The technical instructions mandate testing for immune reactivity to *M. tuberculosis* by an interferon-gamma release assay (IGRA) or, before October 2018, by a tuberculin skin test (TST), for children aged 2–14 years in countries with ≥20 TB disease cases per 100,000 population and for persons (of any age in any country) known to have had contact for a prolonged period with a person with smear- or culture-positive TB disease. A person who has a positive TST or IGRA result but chest radiographs that do not suggest TB disease and no other signs or symptoms of disease or known HIV infection meets criteria for class B2 TB, LTBI evaluation. As such, EDN notifies the health department in the jurisdiction where the newly arriving person reports intending to live to facilitate a postarrival follow-up evaluation. A previous analysis of EDN data demonstrated that children who had a positive TST during their overseas examination were frequently (64%) retested during their postarrival evaluations and often received negative results; when retested by TST in the United States, 37% had a negative TST result, and when retested by IGRA in the United States, 74% had a negative IGRA result (Z Wang, MS, CDC, personal communication, November 2021). The high proportion of negative results when retested by IGRA likely reflects cross-reactivity to bacille Calmette-Guerin (BCG) vaccination (common in many immigrant and refugee populations) producing false-positive results in the initial overseas TST. That analysis also showed that the proportion who accepted treatment was higher after a positive IGRA retest (76%) than a positive TST retest (61%) (Z Wang, MS, CDC, personal communication, November 2021). In response, the 2018 technical instructions were changed from allowing testing by TST or IGRA overseas to allowing IGRA only (except in countries where no IGRAs are licensed). This report shows that, among children who met criteria for class B2 TB, LTBI evaluation, and completed a postarrival domestic evaluation, the proportion who received a domestic diagnosis of LTBI increased from 53% (or lower) for each of the previous 5 years to 62% in 2019, consistent with the change to IGRA only overseas. This change is expected to reduce the amount of unnecessary follow-up care required by state health departments while increasing the number of persons treated.

### Syphilis

Syphilis remains a major public health threat, and infection rates are increasing in the United States (*34*). Screening overseas has remained consistent since the early 1990s, requiring first a nontreponemal serologic test (either the rapid plasma reagin or Venereal Disease Research Laboratory test) and, if positive, a treponemal test performed on the same blood specimen for confirmation. A 2014 update to the technical instructions required panel physicians to identify the stage of disease, and a 2017 update expanded the available list of confirmatory tests that panel physicians may use. Compared with primary and secondary syphilis rates reported by age group for the U.S. general population in 2018 (*34*), the findings in this report indicate that rates were lower for refugees and eligible others aged 15–19, 20–24, and 25–29 years, similar to rates among those aged 30–34 years; more than twice as high as rates among those aged 35–39 and 40–44 years; and more than five times as high as rates among those aged 45–54, 55–64, and ≥65 years. In other words, among younger adults, primary and secondary syphilis rates were lower among refugees and eligible others than among similarly aged persons in the U.S. general population; among older adults, the opposite was observed. This phenomenon could reflect a bias in the United States toward selecting for testing those perceived as most at risk (younger adults), leaving older adults undertested, whereas all refugees and eligible others are tested regardless of age. However, the absolute number of primary and secondary syphilis cases detected annually by universal overseas screening of refugees and eligible others aged >14 years is small, ranging from one case in 2019 to 24 cases in 2015.

### Gonorrhea

Gonorrhea is the second most common sexually transmitted infection in the United States (*34*). Historically, panel physicians relied on the medical history and physical examination findings to diagnose gonorrhea. However, because many infections are asymptomatic, in 2016, CDC issued technical instructions that require laboratory testing. Panel physicians perform a nucleic acid amplification test, typically with a urine sample. Compared with gonorrhea rates reported by age group for the U.S. general population in 2018 (*34*), rates for refugees and eligible others were lower or similar for every 5-year age group among persons aged 15–44 years, three times higher among those aged 45–54 years, and six times higher among those aged 55–64 years, similar to the pattern observed for primary and secondary syphilis. However, even among refugees and eligible others, rates were highest among younger adults (15–34 years), ranging from 289 to 668 cases per 100,000 persons with test results, and lowest among adults aged ≥35 years, ranging from 0 to 234 cases per 100,000 persons with test results. Young adults remain at greatest risk for gonorrhea. Overall, the number of gonorrhea cases identified by the addition of laboratory testing is small, with 58 cases detected in 2018 and 73 cases in 2019.

### Hansen’s Disease

Hansen’s disease is rare among refugees and eligible others. However, this disease still occurs in many places, including within pockets of the United States (*35*). In 2014, the technical instructions clarified the need to follow World Health Organization (WHO) treatment regimens for infected persons. In addition, because patients are considered noninfectious after at least 7 days of therapy, according to the WHO protocol (*36*), immigrants, refugees, and eligible others are allowed to travel while receiving therapy to the United States. EDN sends a separate notification to the U.S. Hansen’s Disease Center in Louisiana for the small number of new arrivals (25 cases in 6 years) admitted with Hansen’s disease.

### Vaccination Program for Refugees

The overseas medical examination exists to satisfy statutory requirements (*4*). However, these required medical encounters also offer an opportunity to introduce voluntary public health interventions. Refugees, unlike immigrants, are not required to receive vaccines before arriving in the United States. When unvaccinated or undervaccinated persons are concentrated together, outbreaks of vaccine-preventable diseases are likely. Outbreaks affecting U.S.-bound refugees overseas have caused preventable illnesses and deaths, led to disease importation and spread in the United States, created costly operational disruptions for the U.S. government resettlement programs, and necessitated costly public health responses by U.S. health agencies (*8*,*37*). The vaccination program for U.S.-bound refugees offers 11 vaccines that help prevent 14 diseases. An important priority, as measles cases increase worldwide, is to offer all refugees at least 1 dose of measles-containing vaccine before they arrive in the United States. First-dose coverage with measles-containing vaccine increased from 49% in 2014 to 96% in 2019, and the majority of those not vaccinated in 2019 had a contraindication that precluded vaccination.

The advantages of administering vaccinations before instead of after resettlement include providing protection in settings of higher risk, when it is most needed; avoiding costly disruptions to resettlement; reducing the risk for disease importation; reducing the number of vaccinations state health departments and resettlement agencies must provide (*38*); and reducing the number of vaccinations needed by refugees during the immediate, and often challenging, postarrival period. A critical component of the vaccination program for refugees is ensuring that the EDN record of vaccines received overseas is available when needed in the United States. A previous analysis showed that the EDN record is routinely available at the follow-up evaluation conducted by state health departments shortly after a refugee arrives but might not be available beyond this point (*39*). In 2015, CDC began working with states to add EDN records to state immunization information systems (IIS), which combine vaccination information from different providers into a single consolidated record. As of 2019, EDN records are transferred to IIS in Colorado, Connecticut, Iowa, Kentucky, Maryland, Massachusetts, Minnesota, Nebraska, New York, Tennessee, and Wisconsin. Supplementing IIS with data from EDN allows states to calculate coverage statistics for refugees more easily and provides a more accurate vaccination history.

### Presumptive Treatment for Refugees

Parasitic infections are among the most common infections worldwide, especially in areas with inadequate sanitation (*40*,*41*). CDC recommends numerous presumptive antiparasitic treatments for refugees, including albendazole for soil-transmitted helminthiases, ivermectin for strongyloidiasis (in countries where *L. loa* is not endemic), and praziquantel for schistosomiasis (*42*). CDC also recommends presumptive treatment for *P. falciparum* infection with artemether-lumefantrine for U.S.-bound refugees from parts of sub-Saharan Africa where malaria is endemic. Refugees originating from areas where malaria is not endemic are unlikely to have asymptomatic or subclinical *P. falciparum* infection and can therefore receive directed treatment, if needed, overseas or in the United States (*43*).

Although the risk for spread from imported infections in the United States is low, untreated helminth infections can persist for decades and cause significant harm to the infected persons. Strongyloidiasis can become serious or fatal when disseminated disease occurs (*44*). Chronic schistosomiasis can have significant clinical consequences, including infertility, liver and kidney failure, and bladder cancer (*45*). Untreated malaria can lead to severe illness and death (*46*). This report shows that the presumptive treatment program provides adequate coverage; other analyses have shown that presumptive treatment of U.S.-bound refugees during the overseas medical examination reduces or eliminates parasitic infections for most U.S.-bound refugees (*47*–*49*) and is cost-effective (*50*). Unlike the vaccination program, which offers vaccination to all refugees, presumptive treatment is only offered in certain countries when the benefit outweighs the risk, considering such factors as the epidemiologic risk for infection, severity of outcomes if untreated, adverse event profile of the chemotherapeutic agent, availability and accuracy of testing after arrival, and access to affordable treatment in the United States.

### Program Improvements

Beginning in 1995, notifications of newly arrived immigrants and refugees were sent to state health departments via the U.S. Postal Service. With the inception of the EDN system in 2008, notifications began to be sent electronically. For refugees and most Iraqi and Afghan special immigrant visa holders, EDN receives overseas medical examination data electronically directly from IOM, the agency that conducts examinations and coordinates travel for most refugees and special immigrant visa holders. For immigrants, collection of overseas medical data historically has involved transferring paper records from immigrants with a medical classification to DHS and then to CDC at U.S. ports of entry and then shipping the paper records to CDC headquarters for manual data entry. This process has taken up to 4 weeks, resulting in delays in the notifications sent to state and local health departments, causing losses to follow-up. In 2018, CDC, in collaboration with DOS, launched the U.S. version of eMedical, a system for processing overseas medical examination data for immigrants. Panel physicians enter data directly into the eMedical system, and the data are transferred to the EDN system within 2 days of the immigrant’s arrival in the United States. The substantial reduction in record-processing time increases the likelihood that health departments will be able to initiate timely follow-up with new-arrival immigrants. Finally, eMedical is used to collect data for all new-arrival immigrants, rather than just for those who arrive with a medical classification.

## Limitations

The findings in this report are subject to at least five limitations. First, by design, the EDN system only collects information for the approximately 10% of immigrants who have an overseas medical classification; thus, DHS data were used to approximate the immigrant denominators. Second, because data transfer for immigrants during the study period primarily relied on staff at ports of entry to correctly review, retain, and route paper forms for each immigrant with a medical classification, human error likely caused some losses, resulting in possible underestimates of immigrants with medical classifications. These two limitations apply only to data for immigrants, not data for refugees or eligible others, and will be remediated once eMedical is fully implemented. Third, although state and local health departments are encouraged and provided incentives to report postarrival examination results to EDN, underreporting might occur; the proportion of immigrants, refugees, and eligible others who completed a postarrival examination might be higher than indicated in this report. Fourth, among U.S.-bound populations, testing for immune reactivity to *M. tuberculosis* is not routinely required for persons aged ≥15 years or for children aged <15 years living in countries where TB incidence is <20 cases per 100,000 population. Thus, some children, many adolescents, and most adults with LTBI are not identified overseas. Immigrants, refugees, and eligible others who are categorized overseas as having no TB classification should not be assumed to have received an LTBI evaluation. Likewise, the overall proportion of persons categorized as class B2, LTBI evaluation, should not be used as a proxy for LTBI prevalence. Finally, persons who choose to emigrate likely differ in many ways from those who choose to stay in their country of origin, whereas refugees typically differ profoundly from fellow citizens in their countries of origin and citizens in their new countries of asylum or resettlement. Thus, caution should be used when extrapolating from data collected for U.S.-bound immigrants in a given country to that country’s general population, and such extrapolations should not be attempted for refugees.

## Conclusion

Rigorous diagnostic testing, data collection, and data transfer from overseas to local public health officials for U.S.-bound mobile populations can be performed on a worldwide scale. The EDN system plays a critical role in meeting CDC’s mission to reduce illness and deaths among immigrants, refugees, and other globally mobile populations and to prevent the introduction, transmission, and spread of communicable diseases into the United States. The surveillance component of EDN provides data to evaluate outcomes from overseas and postarrival medical examinations, identify areas for improvement, and guide evidence-based recommendations. The findings also demonstrate that the overseas medical encounter can be used to successfully provide new public health interventions. The programmatic component of EDN provides the framework to ensure continuity of care for new arrivals and facilitates domestic public health programming. Finally, EDN provides the means for new or urgent overseas interventions to be quickly documented and communicated transnationally; for example, although not included during the period covered by this report, documentation of testing for and vaccination against SARS-CoV-2 in U.S.-bound populations could be readily achieved.
